# Boosting high-intensity focused ultrasound-induced anti-tumor immunity using a sparse-scan strategy that can more effectively promote dendritic cell maturation

**DOI:** 10.1186/1479-5876-8-7

**Published:** 2010-01-27

**Authors:** Fang Liu, Zhenlin Hu, Lei Qiu, Chun Hui, Chao Li, Pei Zhong, Junping Zhang

**Affiliations:** 1 Department of Biochemical Pharmacy, School of Pharmacy, Second Military Medical University, Shanghai 200433, China; 2 School of Life Sciences & Biotechnology, Shanghai Jiao Tong University,China; 3 Department of Mechanical Engineering and Materials Science, Duke University, Box 90300, Durham, NC 27708-0300, USA

## Abstract

**Background:**

The conventional treatment protocol in high-intensity focused ultrasound (HIFU) therapy utilizes a dense-scan strategy to produce closely packed thermal lesions aiming at eradicating as much tumor mass as possible. However, this strategy is not most effective in terms of inducing a systemic anti-tumor immunity so that it cannot provide efficient micro-metastatic control and long-term tumor resistance. We have previously provided evidence that HIFU may enhance systemic anti-tumor immunity by *in situ *activation of dendritic cells (DCs) inside HIFU-treated tumor tissue. The present study was conducted to test the feasibility of a sparse-scan strategy to boost HIFU-induced anti-tumor immune response by more effectively promoting DC maturation.

**Methods:**

An experimental HIFU system was set up to perform tumor ablation experiments in subcutaneous implanted MC-38 and B16 tumor with dense- or sparse-scan strategy to produce closely-packed or separated thermal lesions. DCs infiltration into HIFU-treated tumor tissues was detected by immunohistochemistry and flow cytometry. DCs maturation was evaluated by IL-12/IL-10 production and CD80/CD86 expression after co-culture with tumor cells treated with different HIFU. HIFU-induced anti-tumor immune response was evaluated by detecting growth-retarding effects on distant re-challenged tumor and tumor-specific IFN-γ-secreting cells in HIFU-treated mice.

**Results:**

HIFU exposure raised temperature up to 80 degrees centigrade at beam focus within 4 s in experimental tumors and led to formation of a well-defined thermal lesion. The infiltrated DCs were recruited to the periphery of lesion, where the peak temperature was only 55 degrees centigrade during HIFU exposure. Tumor cells heated to 55 degrees centigrade in 4-s HIFU exposure were more effective to stimulate co-cultured DCs to mature. Sparse-scan HIFU, which can reserve 55 degrees-heated tumor cells surrounding the separated lesions, elicited an enhanced anti-tumor immune response than dense-scan HIFU, while their suppressive effects on the treated primary tumor were maintained at the same level. Flow cytometry analysis showed that sparse-scan HIFU was more effective than dense-scan HIFU in enhancing DC infiltration into tumor tissues and promoting their maturation *in situ*.

**Conclusion:**

Optimizing scan strategy is a feasible way to boost HIFU-induced anti-tumor immunity by more effectively promoting DC maturation.

## Introduction

In recent years, high-intensity focused ultrasound (HIFU) has emerged as a new and promising treatment modality for a variety of cancers, including breast[1], prostate[2], kidney, liver[3], bone[4], uterus and pancreas cancers[5,6]. By focusing acoustic energy in a small cigar-shaped volume inside the tumor, HIFU can rapidly raise the tissue temperature at its beam focus above 65°C, leading to cellular coagulative necrosis and thermal lesion formation in a well-defined region. In principle, HIFU can be applied to most internal organs with an appropriate acoustic window for ultrasound transmission except those with air-filled viscera such as lung or bowel. In particular, HIFU is advantageous in treating patients with unresectable cancers, such as pancreatic carcinoma, or with poor physical condition for surgery. Unlike radiation and chemotherapy, HIFU can be applied repetitively without the apprehension of accumulating systemic toxicity. This unique feature allows multiple HIFU sessions to be performed if local recurrence occurs. Clinical studies have already demonstrated promising outcome of HIFU treatment for several types of malignances, including prostate cancer, breast cancer, uterine fibroids, hepatocellular carcinomas, and bone malignances [7,8]. Although some thermal (skin burn, damage to adjacent bones or nerves) and non-thermal (pain, fever, local infection, and bowel perforation) complications of HIFU treatment have been reported, most of the complications were minor and without severe adverse consequences[8,9].

At present, the primary drawback of HIFU is that it cannot be used to kill micro-metastases outside the primary tumor site. In fact, distant metastasis is a major cause of mortality following clinical HIFU therapy[10]. Lengthy treatment time also represents a limitation. Because each HIFU pulse generally creates an ablated spot of ~10 × 3 × 3 mm in size, up to 1000 lesions may need to be packed closely together during HIFU treatment by scanning the beam focus in a matrix of positions to cover entire tumor volume. With current treatment algorithms, this may translate into a procedure time exceeding 4 hours. Currently, the conventional HIFU treatment protocol in clinic utilizes a dense scanning pattern to eradicate as much tumor mass as possible. Nevertheless, local recurrence of the tumor, due to incomplete tissue necrosis, is still frequently observed following HIFU therapy[10,11]. Clearly, the quality and effectiveness of HIFU cancer therapy need further improvement.

In addition to direct localized destruction of tumor tissues, preliminary evidence from several recent studies has suggested that HIFU may enhance host systemic anti-tumor immunity[12,13]. Although the underlying mechanism is still largely unknown, the potential for a HIFU-elicited anti-tumor immunity is attractive and may help to control local recurrence and distant metastasis following thermal ablation of the primary tumor. On the other hand, the anti-tumor immune response reported in previous studies was not strong enough to achieve a therapeutic gain. As mentioned above, local tumor recurrence and distant metastasis are often the cause of failure for HIFU therapy[10,12], indicating the need to augment the host anti-tumor immunity. Therefore, the optimized strategies that can reduce the primary tumor mass and elicit simultaneously a strong anti-tumor immune response are highly desirable.

The induction and maintenance of an effective antitumor immune response is critically dependent on dendritic cells (DCs), the most effective antigen-presenting cells (APCs) that capture antigens in peripheral tumor tissues and migrate to secondary lymphoid organs, where they cross-present the captured antigens to T cells and activate them[14]. To act as potent APCs, DCs must undergo maturation, a state characterized by the upregulation of MHC and costimulatory molecules and the production of cytokines such as IL-12. However, the requisite signals for DC maturation are often absent from the bed of poorly immunogenic tumors, and many tumor cells even actively produce immunosuppressive cytokines such as VEGF to suppress DC function[15]. Thus, DCs infiltrated in tumor tissues typically exhibit a ''suppressed'' phenotype, and show significantly reduced ability to stimulate allogeneic T cells when compared with normal DCs. Such alterations in DCs development and function are associated with tumor escape from immune-mediated surveillance[16,17]. On the other hand, several studies have demonstrated that dying tumor cells responding to chemotherapy or radiotherapy can express 'danger' and 'eat me' signals such as heat-shock proteins (HSPs) on the cell surface or release intracellular HSP molecules to stimulate DCs to mature and elicit a strong anti-tumor immune response[18]. In the setting of HIFU therapy, we have demonstrated *in vitro *that HIFU treatment results in the release endogenous immunostimulatory factors from tumor cells and stimulates DCs to mature[19]. We have further provided evidence that HIFU can stimulate DCs to infiltrate into tumor tissues, migrate to draining lymph nodes after being activated, and subsequently elicit tumor-specific CTL responses[20]. Based on these observations, we have postulated that *in situ *activation of DCs inside HIFU-treated tumor tissue may constitute an important mechanism for HIFU-induced anti-tumor immunity. Given the central role of DCs maturation in the development of anti-tumor immune response, it is reasonable to speculate that an optimized HIFU strategy that can more effectively activate DCs to mature should have potential to elicit a stronger anti-tumor immunity.

The present study was conducted to search for a feasible way to boost HIFU-induced anti-tumor immunity by more effectively stimulating DCs to mature. To this end, we set up an experimental HIFU system and performed a series of tumor ablation experiments in subcutaneous implanted MC-38 and B16 tumor models. We found that the infiltrated DCs were mostly recruited to the periphery of thermal lesions after HIFU exposure and the tumor cells at the periphery of HIFU-induced thermal lesions could more effectively stimulated DCs to mature. Based on these finding, we further hypothesize a sparse-scan strategy that can produce separated thermal lesions and reserve surrounding peripheral tumor tissue may provide more stimuli for DC maturation than currently used dense-scan strategy, and finally enhance the strength of HIFU-induced systemic anti-tumor immune response. By comparing the tumor ablation efficiency and anti-tumor immune response elicited by two different HIFU treatment strategies, i.e., spare vs. dense scan, in well-controlled animal experiments, we demonstrated that it is actually feasible to boost HIFU-induced anti-tumor immunity through optimizing HIFU scan strategy. Finally, we did *ex vivo *experiments to assess the number of tumor-infiltrating DCs and their maturation status in HIFU-treated tumor tissues and found that sparse-scan HIFU was more effective than dense-scan HIFU in enhancing infiltration of DCs into tumor tissues and promoting their maturation *in situ*.

## Materials and methods

### Cell culture

MC-38 mouse colon adenocarcinoma tumor cell line was kindly provided by Dr. Timothy M. Clay of Duke Comprehensive Cancer Center, Duke University (Durham, NC, USA). B16 mouse melanoma cell line and EL4 mouse lymphoma cell line were obtained from Shanghai Institute of Cell Biology and Biochemistry (Shanghai, China). All of cell lines were maintained in complete Dulbeco's modified eagle medium (DMEM), supplemented with 10% fetal bovine serum (FBS) (Gibco, USA) at 37°C and 5% CO_2_.

### Experimental animals and Tumor Model

C57BL/6 female mice, 5-8 weeks old, were purchased from Shanghai SLAC Laboratory Animal CO. LTD (Shanghai, China). Tumor models were prepared by subcutaneously injecting 5 × 10^5 ^MC-38 or B16 tumor cells suspended in 50 μl of PBS in the left hindlimb of the C57BL/6 mice. The tumor was allowed to grow for 8 days to reach a diameter of 8-10 mm before HIFU treatment. All procedures involving animal treatment and their care in this study were approved by the animal care committee of the Second Military Medical University in Shanghai in accordance with institutional and Chinese government guidelines for animal experiments.

### HIFU Exposure System

**Figure 1:**
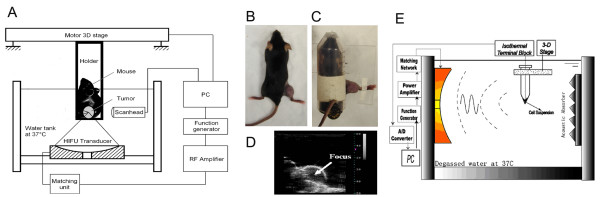
**The experimental HIFU system**. (A) Diagram of the *in vivo *HIFU exposure setup. (B) A tumor-bearing mouse. (C) The way the mouse was fixed during HIFU exposure. (D) The B-mode ultrasound image of the tumor. (E) Diagram of the *in vitro *HIFU exposure setup.

*In vivo *HIFU treatment of tumor was carried out utilizing a B-mode ultrasound imaging-guided HIFU exposure system as reported in our previous study [20] (Figure 1A). A HIFU transducer (provided by Shanghai A&S Science Technology Development CO., LTD, Shanghai, China) with a focal length of 63 mm, operated at 3.3 MHz was mounted at the bottom of a tank filled with degassed water. The transducer was driven by sinusoidal signals produced by a function generator connected in series with a 55-dB power amplifier (DF 5857, Ningbo Zhongce Dftek Electronics Co. Ltd, Ningbo, China). The operation and exposure parameters of the HIFU system were controlled by LabView programs via a GPIB board installed in a PC. During the experiment, the anesthetized animal was placed in a custom-designed holder (Figure 1B and 1C) connected to a 3-D positioning system driven by computer-controlled step motors (provided by Shanghai A&S Science Technology Development CO., LTD, Shanghai, China). To facilitate alignment of the tumor to the HIFU focus, a portable ultrasound imaging system (Terason 2000, Terason, Inc., Burlington, MA) with a 5/10 MHz probe was used to provide B-mode images of the tumor cross section. The medial plane of the tumor was aligned with the focus of the HIFU transducer. Figure 1D shows an example of the B-mode ultrasound images of the tumor grown in the hindlimb of the mouse. As shown in the figure, the tumor outline was clearly defined, with the focus of the HIFU transducer highlighted with a cross-hair indicator. Treatment of the tumor was accomplished through progressive scanning of the whole tumor volume point-by-point, translating the tumor-bearing mouse incrementally with the 3-D step motor positioning system.

*In vitro *HIFU treatment of tumor cells was performed in a HIFU exposure system shown in Figure 1E. The HIFU transducer was mounted horizontally inside a water tank filled with degassed water. 1 × 10^5 ^tumor cells suspended in 20 μl DMEM were loaded in a 0.2 ml PCR thin-walled tube, which was placed vertically with its conical bottom aligned within beam focus of the HIFU transducer.

### Measurement of temperature profile

The temperature profile at the HIFU focus was measured by using a Digital Thermometor (MC3000-000, Shanghai DAHUA-CHINO Instrument Co, Ltd, Shanghai, China) with 0.1 mm bare-wire thermocouple inserted into the tumor tissue or the cell suspension. The thermocouple embedded in the tumor or cell suspension was first aligned to the HIFU focus then temperature elevations and distributions around the center of focus during HIFU exposures were recorded.

### Assay of DC infiltration inside tumor tissue by immunohistochemistry

One day after the HIFU treatment, tumors were surgically excised, freshly frozen in Tissue-Tek O.C.T. compound (Sakura Finetek, Torrance, CA USA), and sectioned at 6 μm thickness. The cryostat sections were then fixed in acetone and immunostained with hamster anti-mouse CD11c mAb (clone HL3, PharMingen). Subsequently, the antibody was visualized using an anti-hamster Ig HRP detection kit (Pharmingen) following the manufacturer's protocol. Finally, sections were counterstained with hematoxylin and evaluated by light microscopy.

### Generation of bone marrow-derived DC [19]

Bone marrow cells were flushed from the femurs and tibiae of female C57BL/6 mice, filtered through a Falcon 100-μm nylon cell strainer (BD Labware), and depleted of red blood cells by five minute incubation in ACK lysis buffer (0.15 M NH4Cl, 1.0 mM KHCO3, 0.1 mM Na2EDTA, pH 7.4). Whole bone marrow cells were plated in six-well plates (BD Labware) in RPMI-1640 supplemented with 10% FCS (GIBCO-BRL, USA), GM-CSF (10 ng/ml), and IL-4 (10 ng/ml) (BD Biosciences Pharmingen, USA), and incubated at 37°C and 5% CO2. Three days later, the floating cells (mostly granulocytes) were removed, and the adherent cells were replenished with fresh medium containing GM-CSF and IL-4. Non-adherent and loosely adherent cells were harvested on day 6 as immature DC (typically contained >90% cells expressing CD11c and MHC class II on the surface, as determined by flow cytometry).

### *In vitro *stimulation of DCs with HIFU-treated tumor cells and assay for their maturation status

5 × 10^5 ^immature DCs generated from mouse bone marrow cells were co-cultured with HIFU-treated B16 tumor cells at ratio of 1:1 in 1 ml of culture for 2 days at 37°C with 5% CO_2_. DC alone, DC stimulated with CpG-ODN1826 (5'-TCCATGACGTTCCTGACGTT-3', Coley Pharmaceutical, Wellesley, MA), which is a known potent DC stimulator, and DC co-cultured with non-HIFU treated B16 tumor cells were used as control. After incubation, supernatants were harvested and assayed for secreted IL-12 and IL-10 by commercial ELISA kits (Biosource International, CA, USA). To analyze the expression levels of co-stimulatory molecules, DCs were collected into cold PBS plus 1% dialyzed bovine serum albumin, then washed and stained on ice for 30 min with a combination of the following monoclonal antibodies: FITC-Conjugated Anti-Mouse CD11c, PE-Conjugated Anti-Mouse CD86, and PE-CY5-Conjugated Anti-Mouse CD80 (BD Biosciences Pharmingen, USA). Subsequently, the cells were washed again and analyzed using a FACSCalibur flow cytometer (Becton-Dickinson).

### Tumor growth regression assay

Following HIFU treatment, Mice were thereafter monitored daily for tumor growth. Mean tumor area for each group was calculated as the product of bisecting tumor diameters obtained from caliper measurements. Measurements were terminated and mice were sacrificed when tumors reached 20 mm in their largest dimension, or when mice became visibly unwell, or when the tumor became ulcerated.

### ELISPOT Assay [20]

Spleens were harvested from euthanized tumor-bearing mice 14 days after HIFU treatment. Splenocytes from mice bearing MC-38 tumors in each group were restimulated in vitro by culture with mitomycin-pretreated MC-38 (specific) or EL4 (irrelevant) tumor cells at 20:1 responder-to-stimulator ratios for 24 h. Splenocytes from mice bearing B16 tumors were stimulated with 1 μg/ml of relevant peptides mouse TRP2_181-188 _(VYDFFVWL, purchased from Dalton Chemical Laboratories Inc. Toronto, ON, Canada), or irrelevant control peptide (OVA_257-264_: SIINFEKL) for 24 h. Re-stimulated splenocytes (1 × 10^6 ^cells in 100 μl medium) were then plated in 96-well nitrocellulose filter plates pre-coated with anti-mouse interferon-γ antibody (Pharmingen, San Diego, CA). After incubation for 24 h at 37°C and 5% CO2, the plates were washed with PBS, and "spots," corresponding to cytokine-producing cells, were visualized by incubation with 100 μl per well of biotinylated antimouse IFN-γ Ab (Pharmingen) overnight at 4°C. After washing with PBS/0.5% Tween, 1.25 μg/ml avidin alkaline phosphatase (Sigma) was added to the well in 100 μl PBS for 1 hour at room temperature. The development of the assay was then performed with l00 μl of 5-bromo-4-chloro-3-indolylphosphate/nitro blue tetrazolium (BCIP/NBT tablets, Sigma) for 10 minutes. The reaction is stopped by the addition of water and the plates allowed drying before counting individual spots with a Zeiss automated ELISPOT reader. The results were expressed as the number of spot-forming cells per 10^6 ^input cells. Overall, three independent experiments were performed with six replicate wells included in each treatment.

### Assay of DC infiltration inside tumor tissue by flow cytometry

One day after the HIFU treatment, tumors were surgically excised. Single cell suspensions were generated from resected tumors as previously described[21]. Briefly, tumors were diced in Ca^2+^- and Mg^2+^-free HBSS after resection, and incubated with 1 mg/ml type IV collagenase (Sigma-Aldrich) for 90 min at room temperature and under constant stirring. EDTA (2 mM) was added to the mixture for 30 additional min before filtration of the cell suspension on 70-μm cell strainers (BD Biosciences). The cell suspension was finally washed twice in HBSS before analysis. For flow cytometry, the following fluorochrome-conjugated antibodies (all purchased from BD PharMingen) were used for staining: CD45-FITC, CD11c-PE, I-A-PE-CY5, CD80-PE-CY5, CD-80-PE-CY5. After adding the appropriate antibody, the cells were incubated at 4°C for 30 min in PBS plus 1% of dialyzed bovine serum albumin and washed twice by centrifugation using fluorescence-activated cell sorting (FACS) buffer. Fluorescence was analyzed with a FACSCalibur flow cytometer and the CellQuest software (Becton-Dickinson).

## Results and Discussion

### HIFU system could produce a typical thermal effect on experimental tumors

**Figure 2:**
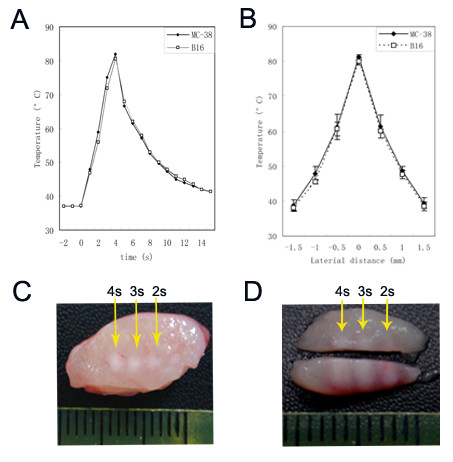
**Thermal effects of HIFU treatment**. (A) Temperature profiles at the beam focus in MC-38 and B16 tumors when the transducer was run in continuous wave (CW) mode at a pressure level of P^+ ^= 19.5/P^- ^= -7.2 MPa. Representative data of three independent experiments with consistent results are shown. (B) Lateral distribution of peak temperature in tumors produced by HIFU during 4-s exposures. Results are expressed as means ± SD out of four independent experiments. (C) Transversal and (D) longitudinal views of thermal lesions produced by HIFU with different treatment duration (4, 3, and 2 s) at above pressure level. The representative section from four treated mice with similar results is shown.

In clinical HIFU therapy, tumor tissue was ablated predominantly by thermal effect which is dependent on the temperature elevation achieved at beam focus during HIFU exposure. If the temperature is raised to 56°C or higher in the tissue, thermal lesion will form within a few seconds as a result of cellular coagulative necrosis. In fact, the temperature within the focal volume may rise rapidly above 80°C during HIFU treatments[22]. In the present study, we at first calibrated our HIFU system to achieve a typical thermal effect on experimental tumors. By adjusting output pressure level and exposure duration, we found that, when the transducer was run in continuous wave (CW) mode at a pressure level of P^+ ^= 19.5/P^- ^= -7.2 (MPa), an elevated temperature was achieved up to 80°C within 4 s at the beam focus in both MC-38 and B16 tumor (Figure 2A). This temperature profile is a representative of the clinical HIFU dosage used in cancer therapy. Under this condition, one HIFU exposure could generate a typical thermal lesion with a well-defined size of 1 × 5 mm (transverse × longitudinal direction) in the treatment region (Figure 2C and 2D). The peripheral tissue around thermal lesion was also heated but with a lower peak temperature (around 55°C) (Figure 2B).

### The infiltrated DCs were mostly recruited to the periphery of thermal lesions after hifu exposure

**Figure 3:**
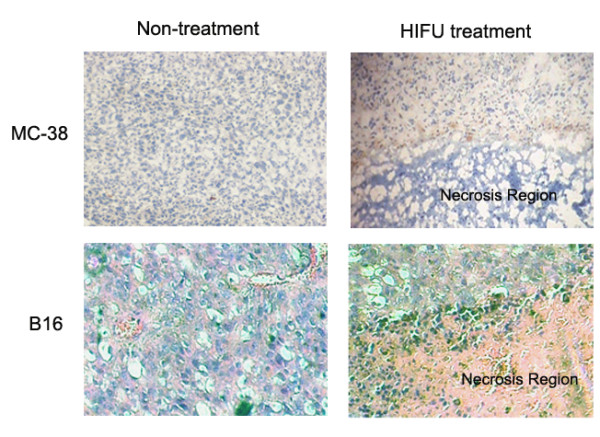
**HIFU-induced DC infiltration surrounding the thermal lesion**. Tumor tissue samples were collected 1 day after HIFU treatment. 6-μm cryostat sections were cut and stained with anti-CD11c Abs. Then the antibody was visualized using the Anti-Hamster Ig HRP detection kit. The sections were counterstained with hematoxylin. Representative sections from each group of four mice are shown.

We next investigated whether HIFU can enhance infiltration of DCs into treated tumor tissues. Tumor samples were collected 1 day after HIFU treatment, and 6-μm cryostat sections were cut and stained with anti-CD11c Abs. Figure 3 showed the results of a representative experiment. In the untreated tumor, only a small amount of DC infiltration was observed. In contrast, DC infiltration was enhanced in HIFU-treated tumor tissues. Most interestingly, it was noted that the infiltrated DC was recruited to the periphery of thermal lesion (Figure 3).

### Tumor cells at the periphery of HIFU-Induced thermal lesion may possess a stronger immunostimulatory property for DCs maturation

**Figure 4:**
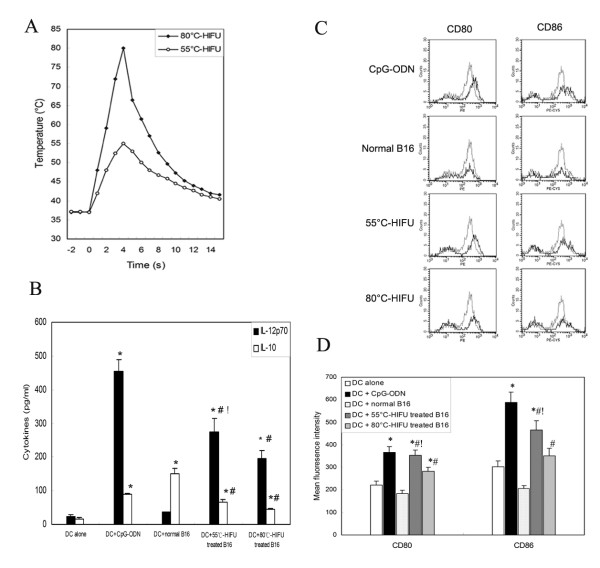
**DC maturation stimulated by HIFU-treated tumor cells**. (A) Temperature profiles produced by 55°C-HIFU and 80°C-HIFU. (B) Immature DCs were incubated for 2 days in the presence of CpG-ODN, normal B16 cells, 55°C-HIFU and 80°C-HIFU treated B16 cells. Levels of IL-12 p70 and IL-10 in the culture supernatants were measured by ELISA. (C) Expression of CD80 and CD86 on the surface of DC (thick line) was assayed by Flow cytometry. Solid thin line represents the expression of these markers on surface of non-stimulated DC. Representative data out of three separate experiments are shown. (D) The expression levels of CD80 and CD86 on DCs were presented as mean fluorescence intensity. Results in panels B and D are expressed as means ± SD out of three independent experiments. * p < 0.05 compared with 'DC Alone', ^# ^p < 0.05 compared with 'DC+normal B16', ^! ^p < 0.05 compared with 'DC+80°C-HIFU' by Student's *t *test.

A prior study has documented a significant up-regulation of HSPs at the border zone of HIFU-induced thermal lesion in patients with benign prostatic hyperplasia[23]. HSPs have been shown to interact with a number of receptors present on the surface of DCs and promote their maturation[24]. These findings imply the possibility that tumor cells at the periphery of HIFU-induced thermal lesion may possess a stronger immunosimulatory property for DCs maturation. The finding in this study that the infiltrated DCs were mostly recruited to the periphery of thermal lesions after HIFU exposure further raises the possibility that tumor cells within this specific zone may have distinct impacts on infiltrated DCs. To provide experimental evidence, we co-cultured immature DCs generated from mouse bone marrow cells with different HIFU-treated tumor cells and assessed their maturing status by assay of IL-12p70/IL-10 production and CD80/86 expression on DCs. We at first determined two different *in vitro *HIFU exposure conditions, under which the temperature in the cell suspension could reach a peak value of 55°C and 80°C respectively within a 4-s exposure duration. Figure 4A showed the distinct temperature profiles in tumor cell suspensions produced by the two different HIFU exposure conditions, which correspond to those produced *in vivo *by HIFU at the periphery and the center of thermal lesion, respectively. For convenience, these exposure conditions were referred to hereafter as "55°C-HIFU" and "80°C-HIFU", respectively. After HIFU treatment, B16 tumor cells were co-culture with immature DC for 2 days, and the release of IL-12p70 and IL-10 and surface expression of maturation markers (CD80 and CD86) on DCs were assayed. DC alone, DC stimulated with CpG-ODN, and DC co-cultured with non-HIFU treated B16 tumor cells were used as control. The results were shown in figure 4B-D. DCs did not spontaneously secrete IL-12p70 and IL-10 when cultured in the absence of exogenous stimuli. CpG-ODN, a known potent DC stimulator, induced the highest level of IL-12p70 production while only moderately increasing IL-10 production, and significantly enhanced the expression of CD80 and CD86, indicating CpG-ODN induced immature DC towards a mature phenotype. Normal B16 tumor cells shown no effects on IL-12 p70 production but markedly increased IL-10 production, and significantly decreased the expressions of CD80 and CD86. Since IL-12p70 and IL-10 are reported as immunostimulatory versus immunosuppressive DC-produced cytokines that may differentially affect the functional outcome of T-cell cross-priming[25,26], this result confirmed previous finding that normal tumors could induce or restrict tumor-infiltrating DCs towards an immature phenotype [16,27]. After HIFU-treatment, however, tumor cells became effective in inducing IL-12p70 production while their effects on IL-10 production markedly reduced. Furthermore, both 55°C-HIFU- and 80°C-HIFU-treated tumor cells significantly enhanced surface expressions of CD80 and CD86 on co-cultured DCs. More importantly, 55°C-HIFU-treated tumor cells showed much more potent immunostimulatory activities than 80°C-HIFU-treated ones, both in the induction of IL-12p70 production and in the upregulation of CD80 and CD86 expression.

Similar results were obtained with the other cell line MC-38 (data not shown). These results demonstrated that HIFU-treatment can change tumor cells from immunosuppressive to immunostimulatory for DCs maturation. More importantly, tumor cells exposed to '55°C-HIFU', which produced a temperature elevation similar to that at the periphery of thermal lesion, exhibited a markedly stronger immunostimulatory potency than those exposed to '80°C-HIFU', which produced a temperature elevation similar to that at the center of thermal lesion. These data therefore provide evidence that tumor cells at the periphery of thermal lesions can more effectively activate DCs to mature than those within the lesions.

We speculated that intracellular HSP molecules release or their membrane exposure induced by HIFU treatments may be the keynote mechanism responsible for the stimulatory activities of DC maturation provided by HIFU-treated tumor cells. We have done some pilot experiments to compare the effects of different HIFU treatments on the expression of HSPs in tumor cells. Our preliminary results suggested the HIFU treatments caused significant up-regulations of HSP70 and HSP90 expression in tumor cells, in which 55°C-HIFU was more effective than 80°C-HIFU (Data not shown). Further studies are underway to determine whether these up-regulated HSPs are released in the extracellular milieu or translocated to cell surface to investigate more deeply the mechanisms of DC activation by HIFU-treated tumor cells.

### It is feasible to boost HIFU-induced anti-tumor immunity through optimizing scan strategy

**Figure 5:**
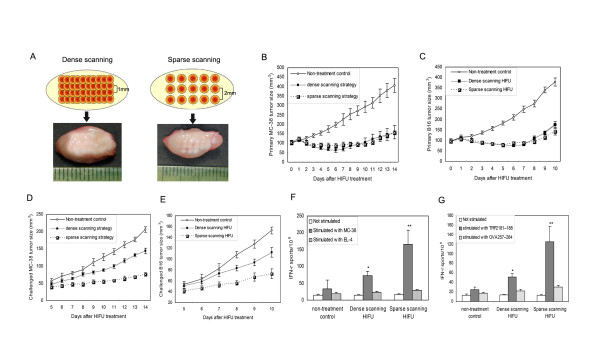
**Comparison of tumor ablation and systemic immune response induced by two different scan strategies**. (A) Thermal lesions produced by dense- and sparse-scan strategies in MC-38 tumors. (B-C) The suppressive effects of different scan strategies on the growth of treated primary tumors. (D-E) The retarding effects on the growth of distant re-challenged tumors. (F-G) Tumor-specific IFN-γ-secreting cells detected in the splenocytes of HIFU-treated mice. C57BL/6 mice were inoculated s.c. on right hind leg with 5 × 10^5 ^MC-38 or B16 tumor cells and treated with different HIFU on day 9 of tumor inoculation. Mice were challenged with 1 × 10^6 ^MC-38 or B16 tumor cells by s.c. inoculation on the left hind leg one day after HIFU treatment. Both primary and challenged tumor growth was monitored daily. Tumor-specific IFN-γ-secreting cells were detected in splenocytes by ELISPOTS assays. Results were expressed as mean ± SD for each group (n = 8 per group). *P < 0.05; **P < 0.001 versus non-treatment control by Student's *t *test. This experiment is representative of three experiments with consistent results.

A dense-scan strategy is usually used in clinical HIFU therapy to produce closely packed or even overlapped thermal lesions to achieve a complete tumor ablation because tumor cells at the board zone of thermal lesion are used to be considered to be heated only sub-lethally and may survive HIFU treatment. However, our data suggest that the presence of such cells *in situ *may lead to "clinical benefit' by potently activating infiltrated DCs to mature. Since the maturation of tumor-infiltrating DCs will lead to the development of strong anti-tumor immunity, an optimized strategy that can reserve these cells in HIFU-treated tumor may have a potential to elicit a stronger anti-tumor immune response. In the clinical setting, the simplest way to achieve this goal is to adjusting the scan strategy, e.g. using a sparse-scan strategy to produce separated rather than closely packed thermal lesions. Hence, we further proposed a sparse-scan strategy may elicit a stronger systemic anti-tumor immune response than currently used dense-scan strategy. To test this hypothesis, we compared the tumor ablation efficiency and anti-tumor immune response elicited by two different HIFU treatment strategies, i.e., sparse vs. dense scan, in well-controlled animal experiments. Because our HIFU system can produced a thermal lesion with a well define size of 1 × 5 mm in the experimental tumor by one pulse of HIFU exposure, a step size of 1 mm was used in dense-scan strategy which can produce closely packed thermal lesions and well mimic the conventional treatment protocol in clinical HIFU therapy. In sparse-scan strategy, the step size was increased to 2 mm to produce a cluster of separated lesions with inter-lesion spacing of 1 mm. Figure 5A showed the closely packed and separated lesions in MC-38 tumor produced by the dense- and sparse-scan strategy, respectively. Tumor growth regression assay revealed HIFU treatment with the sparse- and dense-scan strategies have similar retarding effects on growth of treated tumors (Figure 5B and 5C), even though the total number of thermal lesions produced by sparse scan strategy is much less than that in dense scan strategy. To further assess whether HIFU treatments could induce a systemic anti-tumor immune response *in vivo*, tumor challenge experiments were performed one day following HIFU treatment by injecting 1 × 10^6 ^MC-38 or B16 cells subcutaneously in the contra lateral hindlimb. As expected, the sparse-scan HIFU was found to have a stronger retarding effect on challenged tumor growth (Figure 5D and 5E). To further quantify the anti-tumor immune response, we evaluated whether HIFU treatment could elicit tumor-specific IFN-γ-secreting cells using ELISPOT assay. Consistent with finding in tumor challenge experiments, splenocytes retrieved on day 14 after tumor inoculation in HIFU-treated mice contained significantly more tumor-specific IFN-γ-secreting cells than that from the control group (Figure 5F and 5G). Taken all together, these results demonstrated that optimization of scan strategy in HIFU treatment can indeed induce a more powerful anti-tumor effect and immune response. Here we only focused on proof of principle, so we did not further optimize the inter-lesion spacing or the total number of lesions for the most effective treatment outcome in the present study.

### Sparse-scan HIFU was more effective than dense-scan HIFU in enhancing infiltration of DCs into tumor tissues and promoting their maturation *in situ*

**Figure 6:**
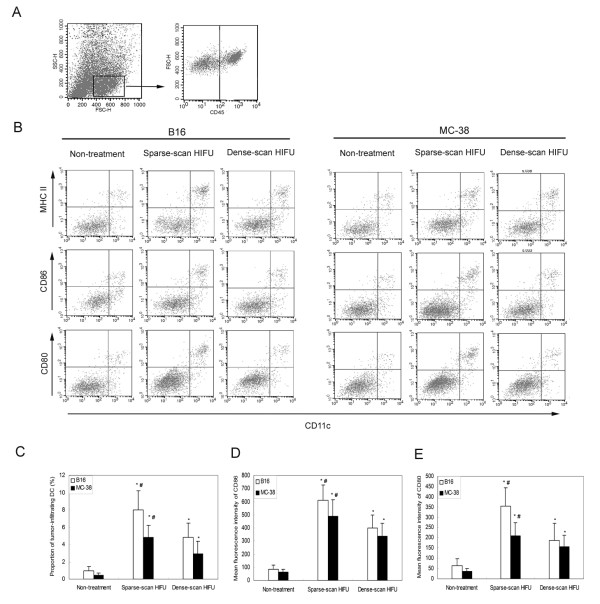
**DCs were recruited into tumor tissues one day after HIFU treatment and exhibited the surface phenotype of maturation**. (A) The presence of CD45^+ ^tumor-infiltrating leukocytes in tumor tissues was identified in the gate indicated. (B) CD11c^+ ^cells in the gate defined in A were analyzed for the expression of MHC II, CD80, and CD86. Representative data of six independent experiments with consistent results are shown. (C) The proportion of tumor-infiltrating DC (CD11c^+^/MHC II^+^) (expressed in percentage of total cells) was investigated for the indicated tumors one day after different HIFU-treatment. (D) The expression levels of CD86 (presented as mean fluorescence intensity) were analyzed in CD11c+ cells infiltrating B16 or MC-38 tumor one day after different HIFU-treatment. (E) The expression levels of CD80 (presented as mean fluorescence intensity) were analyzed in CD11c^+ ^cells infiltrating B16 or MC-38 tumor one day after different HIFU-treatment. (C-E) Results were expressed as mean ± SD for each group (n = 6 per group). *P < 0.05 versus non-treatment control; ^#^P < 0.05 versus Dense-scan HIFU by Student's *t *test. This experiment is representative of three experiments with consistent results.

In order to provide more experimental evidence that the boosted antitumor immune response by sparse-scan HIFU is associated with the stage of the maturation of DCs recruited to the treated tumor, we next determined whether different HIFU treatment could differentially alter DC numbers in the tumor tissues and their functional status. We treated C57BL/6 mice bearing B16 or MC-38 tumors in the left hindlimb with HIFU under sparse- or dense-scan strategy. On the day following HIFU treatment, mice were sacrificed. Upon tumor dissociation, single cell suspensions were generated from resected tumors. The presence of cells with a DC phenotype and their surface expression of the activation markers MHC class II (MHC II), CD80, and CD86 were analyzed by flow cytometry after immunostainning. Leukocytic cells (CD45^+^) could be distinguished by FACS analysis from malignant cells by their size (FSC-H) and morphology (SSC-H) (Figure 6A). In the leukocytic cell population, CD11c^+^/MHC II^+^, CD11c^+^/CD86^+^, and CD11c^+^/CD80^+ ^cells were visualized (Figure 6B), indicating the presence of cells with a DC phenotype. Notably, higher proportion of tumor-infiltrating DCs (CD11c^+^/MHC II^+ ^cells) were recovered from HIFU-treated tumors, as compared with non-treatment control (Figure 6C and 6B), indicating that HIFU-treatment enhanced DC infiltration into tumor tissues. Furthermore, up-regulated levels of the maturation markers CD80 and CD86 were found in CD11c+ subpopulation recovered from HIFU-treated tumors (Figure 6B, D, and 6E), indicating HIFU-treatment induced infiltrated DCs towards a mature phenotype. Most importantly, Sparse-scan HIFU was found to be more effective than dense-scan HIFU in enhancing infiltration of DCs into tumor tissues and promoting their maturation *in situ*, as evidenced by higher proportion of tumor-infiltrating DCs and their higher levels of surface maturation markers (Figure 6B-E). These results suggest that the enhanced antitumor immune response induced by sparse-scan HIFU may be associated with more DC infiltration into treated tumors and effective DC maturation *in situ*.

## Conclusion

In the present study, our results showed that a sparse-scan strategy in HIFU ablation of tumor produced separated thermal lesions with a proper intra-lesion space and elicited a stronger systemic anti-tumor immune response than currently used dense-scan HIFU strategy, which is associated with more effective promotion of DC maturation by tumor cells at the periphery of thermal lesions.

These preliminary findings have significant implications for improving HIFU treatment of cancer in clinic. The conventional treatment protocol in clinical HIFU therapy utilizes a dense-scan strategy to produce closely packed or overlapped thermal lesions aiming at eradicating as much tumor mass as possible. Although effective in reducing the primary tumor mass, this strategy is time consuming and is not most effective in terms of induction of systemic anti-tumor immunity, so that it can not provide efficient micro-metastatic control and long-term tumor resistance for cancer patients. Here we proposed that, by simply adjusting scan strategy to produce separated thermal lesions with a proper intra-lesion space, a stronger systemic anti-tumor immune response may be elicited while not impair its tumor ablation efficiency, so that the overall quality and effectiveness of HIFU cancer therapy can be improved and the treatment time can be significantly shorten. Future clinical studies will tell whether this promise comes true.

## Competing interests

The authors declare that they have no competing interests.

## Authors' contributions

FL and ZH conducted the study, participated in data interpretation, performed the statistical analysis, and drafted the manuscript. LQ participated in the *in vitro *studies. CH and CL participated in the *in vivo *studies. PZ and JP participated in design, coordination, and data interpretation and drafted the manuscript. All authors read and approved the final manuscript.
